# *Fusobacterium nucleatum* and *Bacteroides fragilis* detection in colorectal tumours: Optimal target site and correlation with total bacterial load

**DOI:** 10.1371/journal.pone.0262416

**Published:** 2022-01-07

**Authors:** Marie S. Rye, Kerryn L. Garrett, Robert A. Holt, Cameron F. Platell, Melanie J. McCoy

**Affiliations:** 1 Molecular Oncology, St John of God Pathology, Perth, Western Australia, Australia; 2 British Columbia Cancer Genome Sciences Centre, Vancouver, British Columbia, Canada; 3 Colorectal Cancer Unit, St John of God Subiaco Hospital, Perth, Western Australia, Australia; 4 Medical School, The University of Western Australia, Perth, Western Australia, Australia; University of California Los Angeles, UNITED STATES

## Abstract

**Background:**

Mucosal infiltration by certain bacterial species may contribute to the development and progression of colorectal cancer (CRC). There is considerable variation in reported detection rates in human CRC samples and the extent to which bacterial infiltration varies across regions of the primary tumour is unknown. This study aimed to determine if there is an optimal site for bacterial detection within CRC tumours.

**Methods:**

Presence of target bacterial species was assessed by quantitative real-time PCR (qPCR) in 42 human CRC tumours. Abundance in primary tumour regions, normal epithelium and at metastatic sites was investigated in an expanded cohort of 51 patients. Species presence/absence was confirmed by diversity profiling in five patients. Correlation with total bacterial load and clinicopathological features was assessed.

**Results:**

*Fusobacterium nucleatum* and *Bacteroides fragilis* were detected in tumours from 43% and 24% of patients, respectively (17% positive for both species). The optimal detection site was the tumour luminal surface (TLS). Patients testing positive at the TLS frequently tested negative at other sites, including central tumour and invasive margin. *F*. *nucleatum* was detected at a higher frequency in tumour versus normal epithelium (p < 0.01) and was associated with more advanced disease (p = 0.01). Detection of both species correlated with total bacterial load. However, corroboration of qPCR results via diversity profiling suggests detection of these species may indicate a specific microbial signature.

**Conclusions:**

This study supports a role for *F*. *nucleatum* in CRC development. Presence of *F*. *nucleatum* and *B*. *fragilis* varies across primary tumour regions, with the TLS representing the optimal site for bacterial detection.

## Introduction

The human body is home to an enormous variety of microorganisms, collectively referred to as the microbiome. While some can be pathogenic, most help to maintain the body’s normal function, for example by aiding digestion and regulating the immune system. The composition of the microbiome varies widely between individuals [1] and the importance of microbiome composition, particularly of the gut, in regulating disease susceptibility and treatment response is becoming increasingly recognised [2–5].

In recent years, there has been mounting evidence implicating *Fusobacterium* in colorectal cancer (CRC) tumorigenesis. *Fusobacterium* is more abundant in stool samples from CRC patients than from healthy controls [6–10] and studies have found an increased presence of this bacteria in tumour tissue compared to matched normal tissue or precursor lesions [7,8,11–16]. Stool and normal mucosa samples from individuals with pre-cancerous adenomas and polyps also harbor an increased abundance of fusobacteria compared to healthy controls, which correlates with inflammatory cytokine levels, suggesting its presence may be a cause rather than a consequence of malignancy [6,17,18]. Consistent with this idea, relative abundance of fusobacteria in tumour samples increases with disease stage and has been associated with poorer survival [7,12,15,19]. *Fusobacterium nucleatum* (*F*. *nucleatum*) is the predominant *Fusobacterium* species associated with CRC development and progression to date [10,13,19]. *In vitro*, *F*. *nucleatum* can invade human CRC cells and promote oncogenesis via activation of β-catenin signaling [12,20]. Attachment of *F*. *nucleatum* to CRC cells is mediated by binding of Gal-GalNAc, a polysaccharide overexpressed in CRC tissue, by the fusobacterial lectin Fap2 [21]. Fap2 also binds the immunohibitory receptor TIGIT (T cell receptor with Ig and ITIM domains), protecting CRC cells from immune attack [22]. The amount of *F*. *nucleatum* in human CRC tumours is inversely associated with T cell infiltration, supporting an association with reduced anti-tumour immunity [23], and *F*. *nucleatum* persistence has been linked to reduced CD8^+^ T cell infiltration and increased risk of recurrence following neoadjuvant chemoradiotherapy for locally advanced rectal cancer [24]. In the Apc^Min/+^ mouse model, in which mice carry an autosomal loss of function mutation in the *adenomatous polyposis coli* (*apc*) gene, animals fed *F*. *nucleatum* develop tumours at an accelerated rate [8].

The *Bacteroides* genus, primarily *Bacteroides fragilis* (*B*. *fragilis*), has also been associated with CRC development. Like *Fusobacterium*, some studies have found *Bacteroides* to be enriched in stool samples from patients with CRC and in tumour tissue versus normal mucosa [6,10,14,25]. However, others have found no difference, or a relative decrease in *Bacteroides* abundance in CRC [9,11,13]. Enterotoxigenic *B*. *fragilis* (ETBF), but not nontoxigenic *B*. *fragilis*, is known to induce tumour formation in the Apc^Min/+^ mouse model [26–28], indicating that any association between this species and CRC development may be specifically related to the presence of *B*. *fragilis* toxin (BFT). Consistent with this, the *bft* gene was found to be expressed at a higher rate in *B*. *fragilis* isolates obtained from both mucosal and stool samples from patients with CRC compared to controls [29,30] and BFT has been shown to activate β-catenin signaling in human CRC cells [31].

*Escherichia coli* (*E*. *coli*) has also been found at increased levels in stool samples from patients with CRC compared to healthy controls or those with benign adenomas, and in CRC tissue compared to matched normal tissue or patients with diverticulitis [6,32,33]. Like *F*. *nucleatum* and EBFT, *E*. *coli* also increases tumour formation in Apc^Min/+^ mice [32] and *E*. *coli* strains producing the genotoxin colibactin cause DNA damage and chromosomal instability in eukaryotic cells [34–36].

Co-occurrence of other anaerobic bacteria, including *Campylobacter*, *Leptotrichia*, *Parvimonas* and *Peptostreptococcus* species has also been noted in human CRC tumours and in the faecal microbiome of patients with CRC [6,16,25,37], and a significantly increased incidence of CRC has been consistently observed in patients with *Streptococcus gallolyticus (*formerly *Streptococcus bovis)*, bacteremia/endocarditis [38–42].

Conversely, other species may protect from CRC development. For example, *Bifidobacterium* and *Ruminococcus* are under-represented in the mucosal and faecal microbiome of patients with CRC compared to healthy controls [6,16,25]. *Bifidobacterium* has been shown to promote anti-tumour immunity and improve efficacy of the immune checkpoint inhibitor anti-PD-L1 in mice [43], and both *Bifidobacterium* and *Ruminococcus* have been associated with good response to anti-PD-1 immunotherapy in cancer patients [44–46]. Intratumoral *Bifidobacterium* may also influence tumour characteristics, with one study suggesting a possible link with signet ring cell carcinoma development [47].

Although many previous studies have used patient stool samples to identify bacterial species identified with CRC development and/or treatment response, the faecal microbiome only partially reflects the mucosal microbiome [7,17,48] and may therefore have limited prognostic and predictive value. In studies using patient tissue samples to identify species of interest, detection frequency has varied enormously, ranging from 8.6% to 87.1% for *F*. *nucleatum* [11,15,23,37,49–51]. While differences in detection methods, sample preparation and diet between cohorts may be responsible for a large part of this variation, tumour sampling methods may also be a contributing factor. All of the above-mentioned species can be detected within normal epithelium, and *Fusobacterium* has even been detected in distant metastases [13,52]. However, it is not known to what extent the presence of specific bacterial species varies across different regions of the primary tumour.

This study aimed to assess variation in species detection across CRC tumours and to determine whether there is an optimal target site for detection of infiltrating bacteria. We assessed the abundance of five bacterial species previously associated with CRC development, progression and/or treatment response (*F*. *nucleatum*, *B*. *fragilis*, *Bifidobacterium breve (B*. *breve)*, *Campylobacter showae (C*. *showae)* and *Leptotrichia buccalis (L*. *buccalis)*) in formalin-fixed paraffin-embedded (FFPE) CRC tumour tissue using targeted quantitative real-time PCR (qPCR). We first screened tumours from 42 patients for each species (hereafter referred to as the screening cohort) and then assessed abundance across different regions of the primary tumour, normal epithelium and at metastatic sites in an expanded cohort of 51 patients (referred to as the site investigation cohort). Target species presence/absence was confirmed by diversity profiling in a subset of five patients. We also investigated whether there was any correlation between species detection, total bacterial load and clinicopathological features.

## Methods

### Sample selection

The initial screening stage was designed as a proof-of-concept study and used DNA previously extracted from FFPE colorectal tumour samples for clinical molecular mutation testing between 2012 and 2016. Forty-eight samples containing sufficient DNA for further analysis, collected from patients who had provided informed consent for use of their samples in research studies, were identified for inclusion in this stage of the study. The site investigation cohort included patients who tested positive for one or more target bacteria species in the screening stage, along with 31 additional patients who underwent colorectal tumour resection within the same time period, had given consent for research involvement, and for whom tumour tissue was available for further analysis. Selection of the additional patients was targeted to ensure representation of a range of clinical disease stages and tumour characteristics, such as mucinous histology and areas of inflammation. Haematoxylin and eosin-stained sections were reviewed by KG and regions of interest marked for further DNA extraction from the corresponding FFPE block. Regions of interest included: normal tissue from the proximal and distal surgical margins of the resection specimen, normal tissue adjacent to the tumour region, the tumour luminal surface, central tumour, mucinous tumour (where applicable), invading margin, sites of inflammation, stroma, lymph nodes containing tumour deposits (involved lymph nodes) and metastatic sites where available (S1 Fig).

The study was approved by St John of God Health Care Human Research Ethics Committee (Ref 956) and conducted in accordance with the Declaration of Helsinki. All patients gave written informed consent for their samples and health information to be used for research purposes.

### DNA extraction

DNA used in the screening stage had been previously extracted from regions of FFPE tumour tissue targeted for maximal tumour content using the QIAamp DNA Mini Kit (QIAGEN, Hilden, Germany) according to the manufacturer’s instructions. DNA used in the site investigation stage was extracted from cores of FFPE tissue taken from the specific regions of interest using the Maxwell RSC DNA FFPE Kit (Promega, Wisconsin US) according to the manufacturer’s instructions. All samples were eluted in AE buffer (QIAGEN), quantified using spectrophotometry and stored at -20°C. To minimise contamination, sections were taken from FFPE blocks using a microtome cleaned with 70% ethanol prior to use and between samples. Cores were taken from regions of interest using sealed, sterile needles.

### Detection of target bacterial species

Primer sequences for *F*. *nucleatum* and the *prostaglandin transporter* (*PGT*) positive control were based on those published by Castellarin *et al*. [12]. Sequences for *B*. *fragilis*, *B*. *fragilis toxin* (*bft*), *B*. *breve*, *C*. *showae* and *L*. *buccalis* were obtained from NCBI and primers designed using Primer Express software (Thermo Fisher Scientific, Massachusetts, US; S1 Table). All primers and a pre-designed β*-actin* reference assay were obtained from Integrated DNA Technologies (IDT), Iowa, US.

Primer specificity was confirmed for all primers by testing against target strain DNA. *L*. *buccalis* (DSM-1135), *B*. *breve* (DSM-20213) and *C*. *showae* (DSM-19458) DNA were obtained from DSMZ (Braunschweig, Germany). *B*. *fragilis* and *F*. *nucleatum (strain 7–1)* DNA samples were kindly provided to us by Dr Emma Allen-Vercoe of the University of Guelph, Ontario, Canada.

Bacterial detection was performed on the ViiA7 Real-time PCR System (Thermo Fisher Scientific) using PrimeTime^®^ qPCR primers, probes and mastermix (IDT) according to the manufacturer’s instructions. Reactions were performed using 1X PrimeTime^®^ Gene Expression Master Mix, 1X PrimeTime^®^qPCR Assay and up to 10ng of DNA. Cycling conditions were 95°C for 3 minutes, 60 cycles of 95°C for 5 seconds and 60°C for 30 seconds. Amplification results were reviewed using QuantStudio^™^ Real-Time PCR Software version 1.1 (Thermo Fisher Scientific). Relative expression is reported as delta Ct (dCt) *PGT* minus target, where a higher value corresponds to higher relative expression.

All qPCR reactions for controls and tests were evaluated in duplicate, except in the follow-up site investigation stage, where *B*. *fragilis* and *F*. *nucleatum* assays were performed in triplicate. In the screening stage, results were reported if one or both samples amplified. In the site investigation stage, results were only reported where two or more positive amplifications were seen and the SD of the replicate Ct values was < 5. All single amplifications in the screening stage were confirmed to be positive in the site investigation stage.

### Assessment of total bacterial load

Total bacterial load was assessed using two sets of primers targeting amplification of 16S rRNA. One set, published by Nadkarni *et al*. [53] were obtained from IDT (referred to as 16S-IDT) and a commercially available set of primers targeting pan-bacteria detection of 16S rRNA (Assay ID Ba04230899_s1; Thermo Fisher Scientific, referred to as 16S-TFS). Amplification of the 16S rRNA was performed on the ViiA 7 and reviewed using QuantStudio^™^.

### Diversity profiling

Diversity profiling was performed by AGRF (Australian Genome Research Facility, Melbourne Australia). Samples were amplified with universal primers to the V1-V3 region of the bacterial 16S gene (forward AGAGTTTGATCMTGGCTCAG; reverse GWATTACCGCGGCKGCTG). Amplicons were indexed using the Nextera XT Index Kit (Illumina, San Diego, CA, USA) followed by Paired End sequencing on a MiSeq next generation sequencer (Illumina). Paired-end reads were assembled by aligning the forward and reverse reads using PEAR1 (version 0.9.5). Primers were identified and trimmed. Trimmed sequences were processed using Quantitative Insights into Microbial Ecology (QIIME 1.8) USEARCH (version 8.0.1623) and UPARSE software. Sequences were quality filtered and sorted by abundance after removal of full-length duplicate sequences. Singletons or unique reads were discarded. Sequences were clustered and then chimera filtered using “rdp_gold” database as reference. Reads were mapped back to Operational Taxonomic Units with a minimum identity of 97% and taxonomy was assigned using the QIIME 1 default classifier, pre-trained against Greengenes database5 (Version 13_8, Aug 2013).

### Statistical analysis

Statistical analyses were performed using SAS version 9.4 (SAS Institute Inc., Cary, NC, USA) and GraphPad Prism version 8.0 (GraphPad software Inc., San Diego, CA, USA). Differences in relative expression of *F*. *nucleatum* and *B*. *fragilis* target genes and 16S rRNA by disease stage and species positivity status were assessed using one-way ANOVA. Differences across sites were assessed using Brown-Forsythe ANOVA with Dunnett’s T3 multiple comparisons test. Correlations were assessed using Pearson correlation analyses. Concordance was assessed using Kendall’s tau-b and Spearman’s Rho. Associations between 16SrRNA relative expression and bacterial species positivity status were analysed using logistic and multinomial logistic regression. Comparison of species positivity status between groups was performed using the Fisher’s exact test. The D’Agostino and Pearson and Shapiro-Wilk normality tests and the Brown-Forsythe test for equal variances were employed to assess data distribution prior to performing statistical analyses. Differences and associations were considered statistically significant where P < 0.05.

## Results

### Patient and tumour characteristics

Of the 48 patients identified for the site investigation stage, six were excluded due to the clinical molecular mutation testing sample containing DNA extracted from a recurrent tumour, rather than the colorectal primary (five from liver metastases and one from an omental deposit), leaving a total of 42 patients. Twenty patients who tested positive for one or more bacterial species were included in the follow up site investigation cohort, along with 31 additional patients. Tumour material for one patient who tested positive in the screening stage was unavailable for use in the site investigation stage. All patients underwent surgery for colorectal adenocarcinoma between 2009 and 2016 at St John of God Subiaco Hospital, Perth, Western Australia. Patient and tumour characteristics are provided in Table 1. The mean age for both cohorts was 68 years and the majority of patients were male. The screening cohort was more heavily skewed towards later stage disease as these were patients who required molecular testing to determine available treatment options. One sample included in the screening stage was a diagnostic biopsy, the remainder were surgical resections. Three patients included in the screening stage had a second synchronous CRC tumour. Both tumours from these patients, along with synchronous tumours from two additional patients, were analysed in the site investigation phase. Data in Table 1 corresponds to the tumour with the highest histological grade in cases where this differed.

**Table 1:** Patient and tumour characteristics.

	Screening cohort (n = 42)	Site investigation cohort (n = 51)^a^
Age, mean (range)	68 (25–90)	68 (27–92)
Sex, n (%)		
Male	25 (59.5)	38 (74.5)
Female	17 (40.5)	13 (25.5)
Tumour location, n (%)		
Right/transverse colon	20 (47.6)	25 (49.0)
Left colon	18 (42.9)	20 (39.2)
Rectum	4 (9.5)	6 (11.8)
AJCC Stage		
Stage I	0 (0.0)	2 (3.9)
Stage II	3 (7.1)	11 (21.6)
Stage III	19 (45.2)	27 (52.9)
Stage IV	20 (47.6)	11 (21.6)
Tumour grade, n (%)		
Low Grade (well/moderately differentiated)	30 (71.4)	31 (60.8)
High grade (poorly differentiated)	10 (23.8)	15 (29.4)
Not reported	2 (4.8)	5 (9.8)
Lymphocytic infiltration, n (%)		
Present, n (%)	28 (66.7)	39 (76.5)
Absent, n (%)	14 (33.3)	12 (23.5)
Lymphovascular invasion, n (%)		
Present, n (%)	29 (69.1)	28 (54.9)
Absent, n (%)	11 (26.2)	20 (39.2)
Equivocal/not reported	2 (4.8)	3 (5.9)
Perineural invasion, n (%)		
Present, n (%)	15 (35.7)	12 (23.5)
Absent, n (%)	27 (64.3)	39 (76.5)
Extramural vascular invasion, n (%)		
Present, n (%)	24 (57.1)	18 (35.3)
Absent, n (%)	18 (42.9)	33 (64.7)
*KRAS/BRAF status*, *n (%)*		
*BRAF* V600E	9 (21.4)	6 (11.8)
*KRAS* mutation^b^	16 (38.1)	8 (15.7)
*NRAS* Q61R	2 (4.8)	2 (3.9)
None detected^c^	15 (35.7)	7 (13.7)
Not tested	0 (0.0)	28 (54.9)

^a^ 20 patients from screening cohort included in site investigation cohort.

^b^ Includes A146T, G12A, G12C, G12D, G12R, G12S, G12V, G13D and G13R.

^c^ Evaluation limited to *KRAS* exon 2 and *BRAF* exon 15 in 9 cases.

### Detection of *F*. *nucleatum* and *B*. *fragilis* in FFPE colorectal tissue

*F*. *nucleatum* was detected in tumours from 18/42 patients (43%) and *B*. *fragilis* in 10/42 patients (24%) in the screening phase. Seven patients (17%) tested positive for both species. *B*. *fragilis* toxin, *B*. *breve*, *C*. *showae* and *L*. *buccalis* were not detected in any of the tumour samples. All samples tested positive for the house-keeping genes *PGT* and β*-actin*.

In the follow-up site investigation phase, a total of 56 tumours were analysed (51 patients, five with synchronous tumours). DNA samples were available for the tumour luminal surface (TLS), normal proximal epithelium and invasive margin for all 56 tumours. Samples representing normal distal epithelium, normal adjacent epithelium and the central tumour were available for 55, 50 and 50 of the 56 tumours, respectively. The tumour stroma was sampled in 36 of the 56 tumours analysed. Site of inflammation and mucinous regions were identified in 31 and 23 of 56 tumours. A total of 34 patients had involved lymph nodes available for analysis and four patients had tissue from loco-regional peritoneal metastases (mesenteric nodule, omentum, fallopian tube and peritoneum).

**Fig 1:**
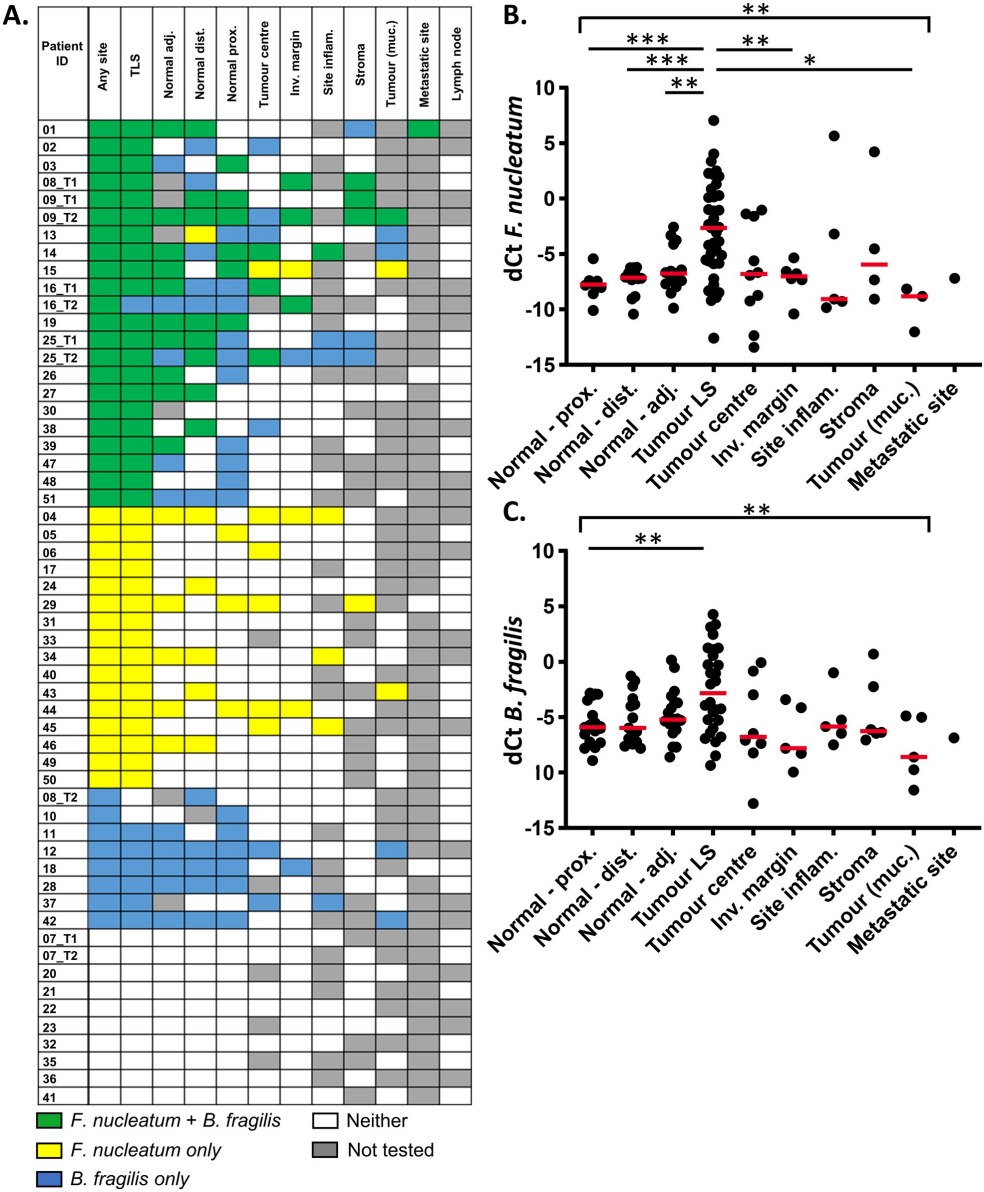
Relative abundance of *F*. *nucleatum* and *B*. *fragilis* by site. (**A**) Heatmap of *F*. *nucleatum* and *B*. *fragilis* status by site for each patient. Samples ordered by species status at any site, sites ordered by species prevalence. T1 and T2; tumours 1 and 2 for patients with synchronous tumours, TLS; tumour luminal surface. (**B** and **C**) dCt (*PGT*—target) for *F*. *nucleatum* and *B*. *fragilis* by region of interest. Dots represent individual tumours, line at median. Groups (excluding metastatic site) compared using Brown-Forsythe ANOVA. Tumour centre and TLS were compared to all other sites using Dunnett’s T3 multiple comparisons test. For patients with synchronous tumours, data for both tumours are shown. ***P<0.001, **P<0.01, *P<0.05.

The optimal site for detection of both *F*. *nucleatum* and *B*. *fragilis* was the TLS (Fig 1). Only one tumour testing positive for *F*. *nucleatum* at any site was negative at the TLS (patient 16 tumour 2; Fig 1A), whereas of the 37 patients testing positive at the TLS, 27 (73%) tested negative at the tumour centre, 32 (86%) tested negative at the invasive margin and 33 (89%) tested negative within the tumour stroma. Of *B*. *fragilis*-positive tumours (n = 28), all were positive at the TLS, but 17 (61%), 23 (82%) and 22 (79%) tested negative at the tumour centre, invasive margin and stroma, respectively.

Comparing detection in tumour versus normal tissue, *F*. *nucleatum* was detected at a significantly higher frequency in the tumour versus the normal epithelium (38/56 *vs* 23/56, P<0.01; Fisher’s exact test). Of the 38 tumours testing positive for *F*. *nucleatum* at any tumour site, 15 (39%) tested negative within the normal epithelium (Fig 1A). Relative abundance was also significantly higher at the TLS compared to the adjacent, distal or proximal normal epithelium; (P<0.01, P<0.001 and P<0.001, respectively (Fig 1B). In contrast, for *B*. *fragilis*-positive tumours, only 2/28 (7%) tested negative within matched normal epithelium (Fig 1A). In two tumours (patient 10 and one of the synchronous tumours from patient 08), *B*. *fragilis* was detected in the normal epithelium, but not at any of the tumour sites. *B*. *fragilis* was also more abundant at the TLS, than within proximal normal epithelium (P<0.01; Fig 1C). The difference between the TLS and adjacent or distal normal epithelium was not statistically significant. Interestingly, abundance of both species at the tumour centre was no higher than within the normal epithelium.

Across the study, both *F*. *nucleatum* and *B*. *fragilis* were detected at least once at all tissue sites tested, with the exception of involved lymph nodes. One of the four loco-regional metastases (the fallopian tube metastasis) tested positive for both bacterial species.

Of the five patients with synchronous tumours, discordant results were observed for one case (patient 08), who tested positive for both species in tumour 1 and neither species in tumour 2 (Fig 1A).

### *F*. *nucleatum* and *B*. *fragilis* detection correlates with total bacterial load

Two sets of primers targeting amplification of 16S rRNA sequences were used to assess total bacterial load in all TLS samples from the site investigation stage and investigate any correlation with *F*. *nucleatum* and/or *B*. *fragilis* detection. Relative PCR amplification of 16S was significantly higher using the 16S-TFS primer set compared to the 16S-IDT primer set. However, data obtained using both primer sets were concordant (Kendall’s tau-b 0.80, P < 0.0001; Spearman’s Rho 0.94, P < 0.0001; S2 Fig). 16S amplification data obtained using the 16S-TFS primers were used for all subsequent analyses, since these data were normally distributed.

**Fig 2:**
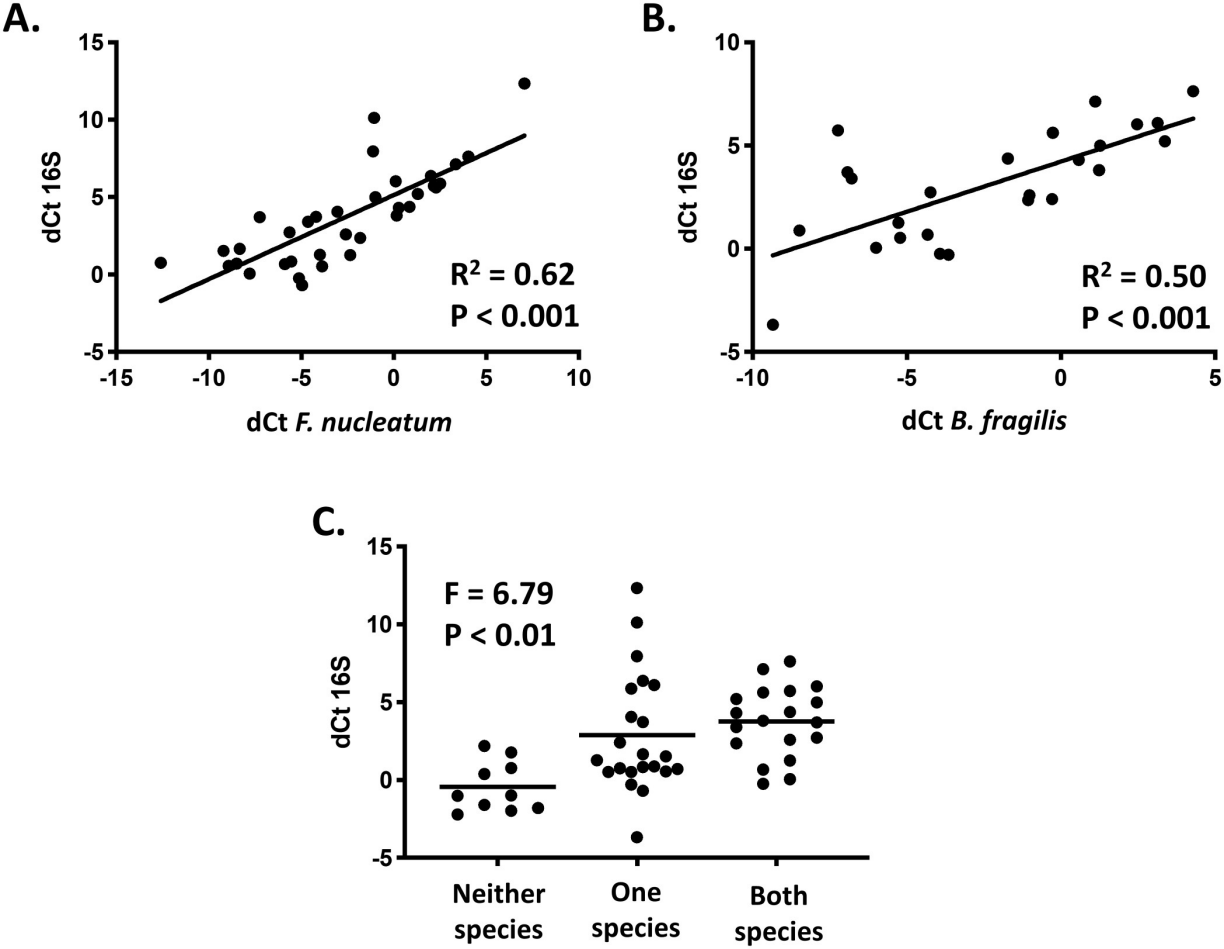
Detection of *F*. *nucleatum* and *B*. *fragilis* correlates with total bacterial load. (**A** and **B**) Pearson correlation analysis of relative *F*. *nucleatum* (**A**) and *B*. *fragilis* (**B**) abundance versus 16S detection (*PGT*—target) at the TLS. (**C**) Relative 16S expression in tumours with neither, one or both species detected, line at mean (ANOVA). For patients with synchronous tumours, data for one tumour is shown (tumour with the highest number of species present in the discordant case). TLS, tumour luminal surface.

There was a strong positive correlation between total bacterial load and relative abundance of both *F*. *nucleatum* and *B*. *fragilis* at the TLS (Fig 2A and 2B). A strong association between total bacterial load and species positivity status was also observed (Fig 2C). Tumours in which relative amplification of 16S rRNA sequences was higher than the median value were more than seven times more likely to be *F*. *nucleatum* positive and almost five times more likely to be *B*. *fragilis* positive compared to tumours with 16S rRNA detection below the median value (odds ratio (OR) 7.3, 95% confidence interval (CI) 1.8–30.7, P = 0.006 and OR 4.8, 95% CI 1.5–15.6, P = 0.01, respectively; logistic regression). Using multinomial logistic regression, tumours with a higher than median total bacterial load were almost 34 times more likely to be positive for both *F*. *nucleatum* and *B*. *fragilis* compared to tumours with a lower than median load (OR 33.7, 95% CI 3.2–351.0, P = 0.003). However, the wide confidence intervals for these differences should be acknowledged.

### *F*. *nucleatum* positivity is associated with more advanced CRC

**Fig 3:**
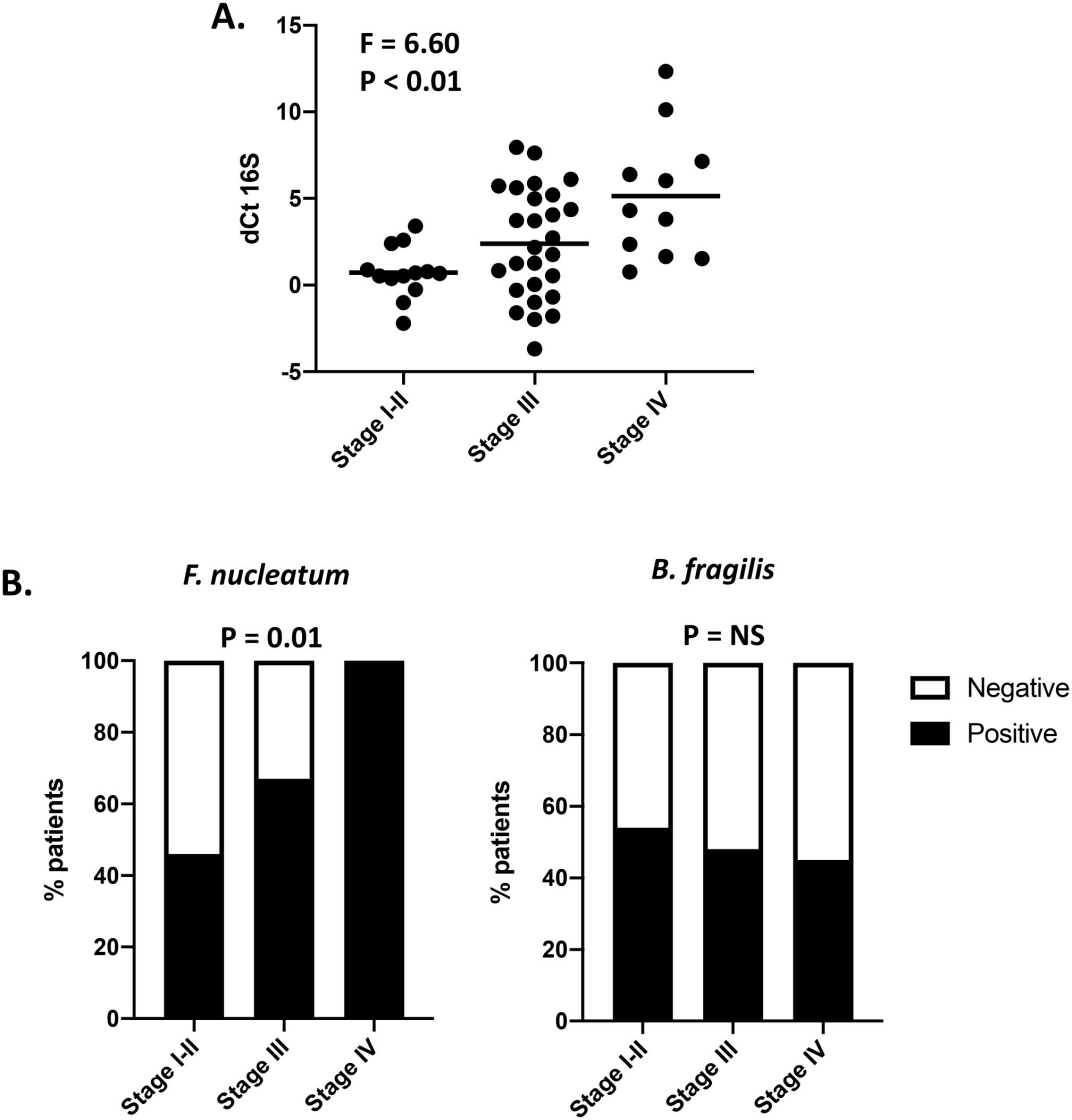
*F*. *nucleatum* positivity is associated with more advanced disease. (**A**) Relative abundance of 16S rRNA (*PGT*– 16S) at the TLS by disease stage, line at mean (ANOVA). (**B**) *F*. *nucleatum* and *B*. *fragilis* positivity status by disease stage (Fisher’s exact test). For patients with synchronous tumours, data for one tumour is shown (tumour with the highest number of species present in the discordant case). TLS, tumour luminal surface.

We then investigated the relationship between total bacterial load/species positivity status and clinicopathological characteristics. There was a strong positive association between bacterial load at the TLS and disease stage (Fig 3A). While a similar trend was observed for *F*. *nucleatum* and *B*. *fragilis* abundance, the associations were not significant (S3 Fig). However, there was a strong relationship between *F*. *nucleatum* positivity status and disease stage, with 100% (11/11) of patients with stage IV disease testing positive for *F*. *nucleatum* at the TLS, compared to 46% (6/13) patients with stage I-II disease and 67% (18/27) patients with stage III disease (P = 0.01, Fisher’s exact test; Fig 3B). No association between *B*. *fragilis* status and disease stage was observed (Fig 3B) and patients with more advanced CRC were not more likely to test positive for both *F*. *nucleatum* and *B*. *fragilis* (S3 Fig). There were no significant associations found between 16S, *F*. *nucleatum* or *B*. *fragilis* relative abundance or species positivity status and patient age, sex, tumour location, histological grade or the presence of *KRAS* or *BRAF* mutations (S2 Table).

### Population diversity profiling

**Fig 4:**
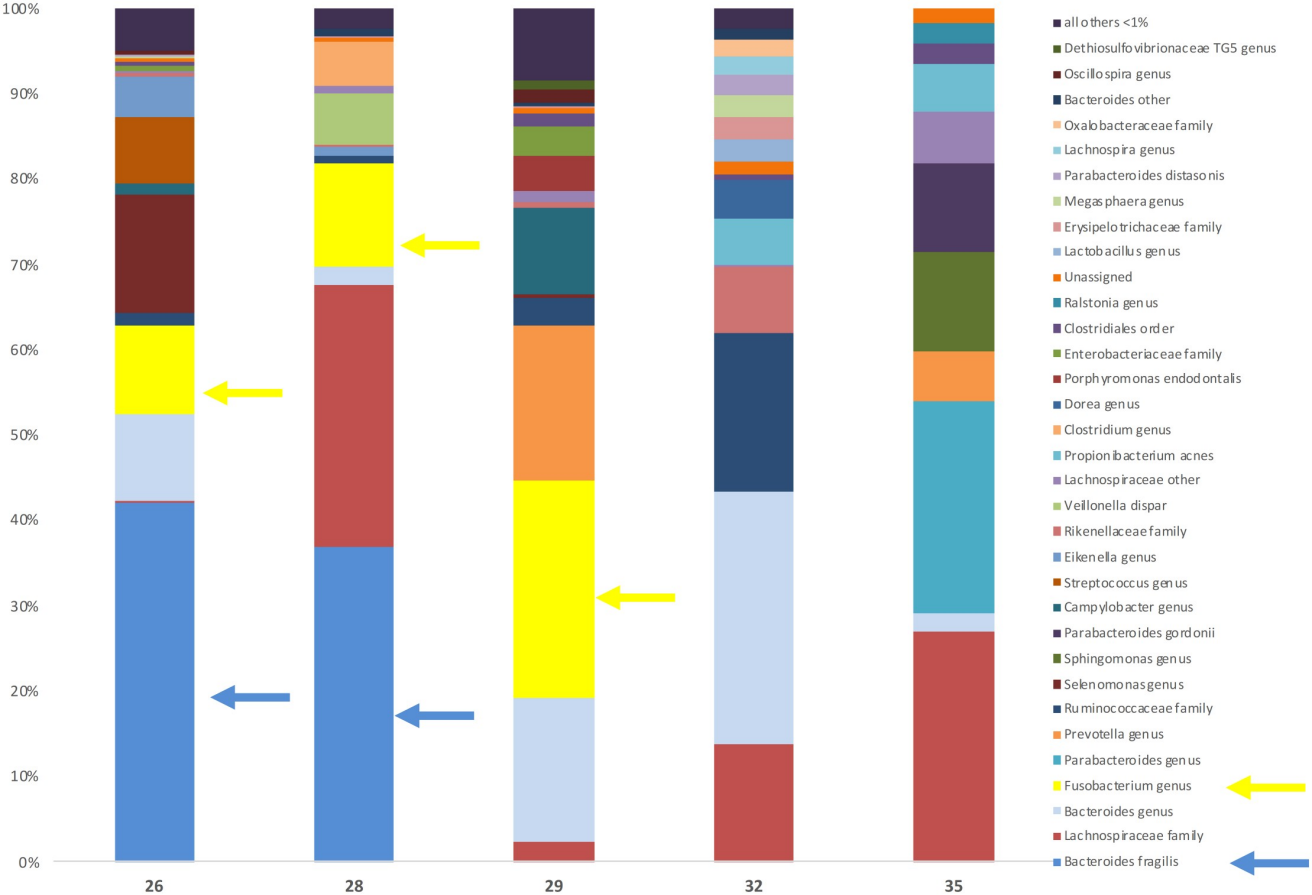
Bacterial composition by diversity profiling. Diversity profiling results for five TLS samples selected based on *F*. *nucleatum* and *B*. *fragilis* positivity status by targeted qPCR (patient 26 was positive for both species, patient 28 was positive for *B*. *fragilis* only, patient 29 was positive for *F*. *nucleatum* only and patients 32 and 35 were negative for both species). *B*. *fragilis* and the *Fusobacterium* genus are highlighted by blue and yellow arrows, respectively.

Five TLS DNA samples were selected to perform a detailed evaluation of overall species diversity and as an orthogonal method to confirm the ability to detect the targeted species by qPCR. Samples were selected based on species positivity status by qPCR (one sample with both species present, one positive for *F*. *nucleatum* only, one positive for *B*. *fragilis* only and two negative for both species), contained between 16 to 88ng/ml total DNA and had given high levels of 16S amplification compared to other samples with the same species positivity status. Due to the strong correlation between 16S and *F*. *nucleatum*/*B*. *fragilis* detection (Fig 2), the samples negative for both species had a comparatively lower level of 16S amplification (mean dCt 2.0 versus 6.2). A summary of the profiling results for all five samples is presented in Table 2 and a detailed breakdown of the reads in Fig 4 and S3 Table.

**Table 2:** Diversity profiling results.

Patient ID	DNA conc. (ng/ml)	dCt 16S	Species by qPCR	Total reads	% reads *Fusobacterium*^a^	% reads *B*. *fragilis*	% reads other *Bacteroides* spp.
26	88.3	6	Both	43,397	10.5	42.1	10.5
28	26.6	6.1	*B*. *fragilis*	34,505	12.1	36.8	3.1
29	70.9	6.4	*F*. *nucleatum*	42,735	25.4	0.0	18.0
32	26.9	2.2	Neither	2014	<0.1	0.0	31.2
35	15.8	1.8	Neither	989	0.0	0.0	2.2

^a^ Results only available at genus level.

Good correlation between targeted qPCR data and broader species profiling was demonstrated in the subset of five samples evaluated (Table 2 and Fig 4). In one case (patient 28) *Fusobacterium* was detected by diversity profiling at 12.1% of the bacterial reads mapped, but *F*. *nucleatum* was not detected by targeted qPCR. The fact that the diversity profiling results were present to the genus rather than species level for *Fusobacterium* may explain this discrepancy. A far smaller fraction of *Fusobacterium* DNA reads (<0.1%) were detected in the sample from patient 32, which was also *F*. *nucleatum* negative by qPCR. At this level it is not surprising that this may not have been detected by the qPCR method.

## Discussion

Mucosal infiltration by *F*. *nucleatum* and other anaerobic bacteria may play a role in the development and progression of colorectal cancer [7–10,12,13,15–18,25,37]. Numerous studies have detected tumour-infiltrating bacteria within human colorectal tumour samples. However, there is considerable variation in reported detection rates of specific species [11,15,23,29,37,49–51,54]. To what extent bacterial infiltration varies across different regions of the primary tumour is poorly understood.

Here we show that *F*. *nucleatum* was frequently detected in tumour samples, with 43% of patients in our screening cohort testing positive. This detection frequency falls in the middle of the range reported in previous studies (8.6%–87.1%) [11,15,23,37,49–51,54]. *B*. *fragilis* was detected in 24% of tumour samples, with 17% of tumours harbouring both *F*. *nucleatum* and *B*. *fragilis*. The optimal site for detection of both species was the tumour luminal surface (TLS), with both species detected at considerably lower frequencies within the tumour centre, at the invasive margin and within the tumour stroma. The higher rate of detection at the TLS likely reflects the presence of bacteria from the lumen of the bowel that have accumulated at the mucosal surface due to abnormal cell growth and compromised cellular architecture. The lack of concordance observed in one of five synchronous tumour pairs suggests that individual tumours may have their own microbial signature, rather than this being host-specific.

Consistent with most previous reports [7,8,12,13,15,16], we detected *F*. *nucleatum* at a higher frequency and relative abundance within tumour samples compared to matched normal epithelium. However, this was only true for the TLS. DNA extracted from material restricted to the tumour centre contained comparable levels of *F*. *nucleatum* DNA to that from the normal mucosa. This may explain the difference between our TLS findings and those of Bundgaard-Nielsen *et al*, who detected *F*. *nucleatum* at a comparable frequency and level in tumour and matched normal tissue using DNA isolated from whole tissue sections [11], where they were more likely targeting the tumour centre region. Our tissue targeting strategy allowed us to be very specific about the region and morphology sampled. We found no difference in frequency of *B*. *fragilis* detection between tumour and normal tissue. This likely reflects a relatively weaker association between *B*. *fragilis* and CRC, which is again consistent with the current literature, with some studies reporting a positive association and others no difference, or a relative decrease in *Bacteroides* abundance in CRC [9,11,13,14]. A clear boundary of morphologically normal cells was present between the tumour and the area targeted as adjacent normal epithelium in this study as we were targeting very small specific regions within the larger paraffin block. Nonetheless, this region may have contained a very small proportion of malignant cells. The presence of incident tumour cells, however, would have made a minimal contribution to the extracted DNA and is therefore unlikely to have significantly impacted the results.

Of the four loco-regional metastases tested, we detected *F*. *nucleatum* in one sample (a fallopian tube metastasis). Of note, this case and two of the three other cases tested positive for *F*. *nucleatum* at the TLS and within the normal epithelium. *F*. *nucleatum* has also been detected in distant CRC metastases, with Bullman *et al*. identifying *Fusobacterium* gene expression in 7/11 liver metastasis samples [52] and Abed *et al*. finding *F*. *nucleatum* within 10/12 liver metastases [21]. In contrast to Yu *et al*., who detected *F*. *nucleatum* in 20/20 lymph node metastases [37], none of the 34 involved lymph node samples we tested yielded positive results. The methods employed by Yu *et al*. differed from the current study, with *F*. *nucleatum* detected by fluorescence in situ hybridization rather than qPCR, which may partially explain the difference in results. Nonetheless, the presence of *F*. *nucleatum* at metastatic sites supports the hypothesis that *F*. *nucleatum* plays a role in CRC progression, which is thought to be mediated, at least in part, by release of pro-inflammatory cytokines including IL-8 and CXCL1 following Fap2-dependent cell invasion [55]. Consistent with this hypothesis, *F*. *nucleatum* positivity correlated with increasing disease stage in this patient cohort, as previously reported [12,15,19]. *F*. *nucleatum* abundance has also been associated with tumour location (increased in proximal versus distal cancers), *BRAF* mutation, high grade histology and microsatellite instability (MSI) [7,15,19,49,51]. We did not find any significant associations between *F*. *nucleatum* presence and specific pathological features, but the study was not powered to detect these and MSI status was not available for this cohort.

It is possible that detection of specific bacterial species is simply a reflection of total bacterial load; the more bacteria present in a sample, the greater the likelihood of detecting the species of interest. We did observe a strong positive correlation between both *F*. *nucleatum* and *B*. *fragilis* relative expression and 16S amplification at the TLS. However, diversity profiling of a small subset of samples indicated wide variation in bacterial species composition and confirmed the presence or absence of our target species as detected by qPCR, suggesting that detection of these species may indicate a specific microbial signature, rather than being solely an indirect measure of bacterial content. The success of diversity profiling increases with bacterial load due to the requirement for sufficient input DNA to obtain suitable libraries. Therefore, it should be acknowledged that the two samples negative by qPCR for both species that were selected for diversity profiling had a lower overall bacterial content. However, these samples provided sufficient mapped reads when sequenced and were deemed suitable for analysis. Since diversity profiling was only performed on TLS samples, we cannot comment on how bacterial diversity may vary across different regions of the primary tumour or how it compares to that within the normal mucosa or faecal microbiome.

The comparable relative abundance of *B*. *fragilis* between tumour and normal epithelium and the lack of association with disease stage observed in this study are consistent with any causal link with CRC development being limited to enterotoxigenic *B*. *fragilis* [26–30]. All DNA samples used in the screening phase of the study were assessed for *bft* gene expression as well *B*. *fragilis gyrase*. However, none returned a positive result for *bft*. Previous studies using CRC samples have assessed *bft* gene presence in *B*. *fragilis* isolates, rather than directly in mucosal tissue [29,30], which may explain the difference between these and our results.

*B*. *breve*, *C*. *showae* and *L*. *buccalis* were not detected in the screening patient cohort. This may be due to a true absence or a level of abundance below that detectable by qPCR in these samples, or due to the choice of primers used. While specificity was confirmed using purified target strain DNA, no extracted DNA samples tested positive for these species and as such we are unable to rule this out. The diversity profiling did report *Campylobacter* to represent >10% of the bacterial population in the TLS sample from patient 29, who was included in the screening cohort. However, profiling data were only available to the genus level and the DNA used in the screening stage was extracted from an area of tissue selected for maximal tumour content, rather than specifically targeting the TLS. The *Leptotrichia* genus was detected at a frequency of 0.46% in patient 29 and was not detected in the other four patients. Data for *Bifidobacteria* were not identified in the diversity profiling results.

A potential limitation of this study is that our DNA extraction protocol was optimized for analysis of human, rather than microbial DNA. This may explain the lack of *B*. *breve* detection by either targeted qPCR or 16S rRNA diversity profiling. *Bifidobacteria* are gram-positive organisms and thus their increased cell wall strength may make standard DNA extractions methods less successful, with mechanical disruption often also required [56]. This may also have impacted our quantitation of total bacterial DNA. However, the 16S diversity profiling did identify some gram-positive species, such as *Propionibacterium*, suggesting that sufficient cell wall disruption may have occurred during the formalin-fixation and paraffin-embedding process to allow access to gram-positive bacterial DNA.

The use of 60 cycles in our qPCR protocol was designed to go well past the threshold Ct. Most samples reported as positive had a mean Ct well below this value (23.0–42.8 (median 37.0) across all samples for both stages or *F*. *nucleatum*, and 24.4–45.9 (median 36.2) for *B*. *fragilis*). However, these data indicate a very low relative abundance of our target species in some of the positive samples. Importantly, samples were only reported as positive where two or more of the three replicates amplified and where the replicates had a SD < 5. In the screening stage, where tests were evaluated in duplicate, single amplifications were reported as positive, and confirmed to be so in the site investigation stage using DNA freshly extracted for the same tumour.

Although tissue processing reagents were changed daily (routine laboratory protocols), microtome and blades were cleaned prior to use and between samples and sterile needles were used to obtain tissue for DNA extraction, it is likely that at least some of the bacteria detected in the samples may be due to contamination. Processing conditions were consistent across samples and confirmation of the presence of target species in screening stage samples using DNA freshly extracted from the same tumours in the site investigation phase, along with the variation in species composition seen by diversity profiling, suggests inherent differences in the bacterial content of the samples. However, it should be acknowledged that any bacterial contamination may have had a larger impact on data obtained from samples with a low endogenous bacterial load.

The 16S diversity profiling component of the study was not intended as a comprehensive bacterial signature analysis, rather as an orthogonal method to corroborate our findings obtained by qPCR. Nonetheless it is important to recognise that taxonomic identification to the species level based on 16S percentage sequence similarity has limitations [57]. The data obtained using OTU clustering with 97% similarity to the highly curated Greengenes database cannot therefore be considered definitive confirmation of the presence of *B*. *fragilis* but is strongly supportive of the targeted species identification using qPCR.

The primary aim of this study was to assess variation in species abundance across different regions of primary CRC tumours, rather than to identify a microbial signature associated with CRC. We therefore selected five species for investigation. We cannot comment on the abundance of other CRC-associated bacterial species in this cohort or whether these are also more prevalent at the TLS.

In summary, we provide further evidence to support a role for *F*. *nucleatum* in CRC. We show that *F*. *nucleatum* and *B*. *fragilis* detection varies significantly according to the region of the primary tumour sampled and identify the tumour luminal surface as the optimal site for detection of these species. This has implications for future studies assessing the abundance of bacterial species in CRC specimens and for understanding the potential mechanisms involved in bacterial-driven disease progression.

## Supporting information

S1 Fig Region of interest selection. Representative images illustrating selection of regions of interest. Areas of proximal and distal normal epithelium were selected from the proximal and distal resection margins, respectively. Scale bars 5mm (left column) and 200mm (centre and right columns).
(PDF)

S2 Fig Comparison of primer sets for assessment of total bacterial load. Relative amplification of 16S rRNA sequences using 16S-TFS primers (Thermo Fisher Scientific) and 16S-IDT primers (Integrated DNA Technologies, as published by Nadkarni et al 2002). (**A**) Groups compared using the Wilcoxon matched pairs test. Line at median. (**B**) Spearman’s Rho correlation analysis.
(PDF)

S3 Fig Abundance of *F*. *nucleatum* and *B*. *fragilis* by disease stage. Relative expression (PGT—target) for *F*. *nucleatum* (**A**) and *B*. *fragilis* (**B**) at the TLS by disease stage, line at mean (ANOVA). **C**. Species positivity status at the TLS by disease stage (negative for *F*. *nucleatum* and *B*. *fragilis* vs single positive for *F*. *nucleatum* or *B*. *fragilis* vs double positive for *F*. *nucleatum* and *B*. *fragilis*), Fisher’s exact test. TLS, tumour luminal surface.
(PDF)

S1 Table Primer details. (PDF)

S2 Table Species positivity status and total bacterial load according to clinicopathologic factors. (PDF)

S3 Table Diversity profiling results. Percentage of total reads attributed to each taxon.
(PDF)
