# Supplementary material for: Fusobacterium nucleatum and Bacteroides fragilis detection in colorectal tumours: Optimal target site and correlation with total bacterial load
Source: PLoS One. 2022 Jan 7;17(1):e0262416. doi: 10.1371/journal.pone.0262416 (PMC8740967; doi:10.1371/journal.pone.0262416)
Supplement: S1 Fig — Representative images illustrating selection of regions of interest. Areas of proximal and distal normal epithelium were selected from the proximal and distal resection margins, respectively. Scale bars 5mm (left column) and 200mm (centre and right columns). (PDF) [file pone.0262416.s001.pdf]

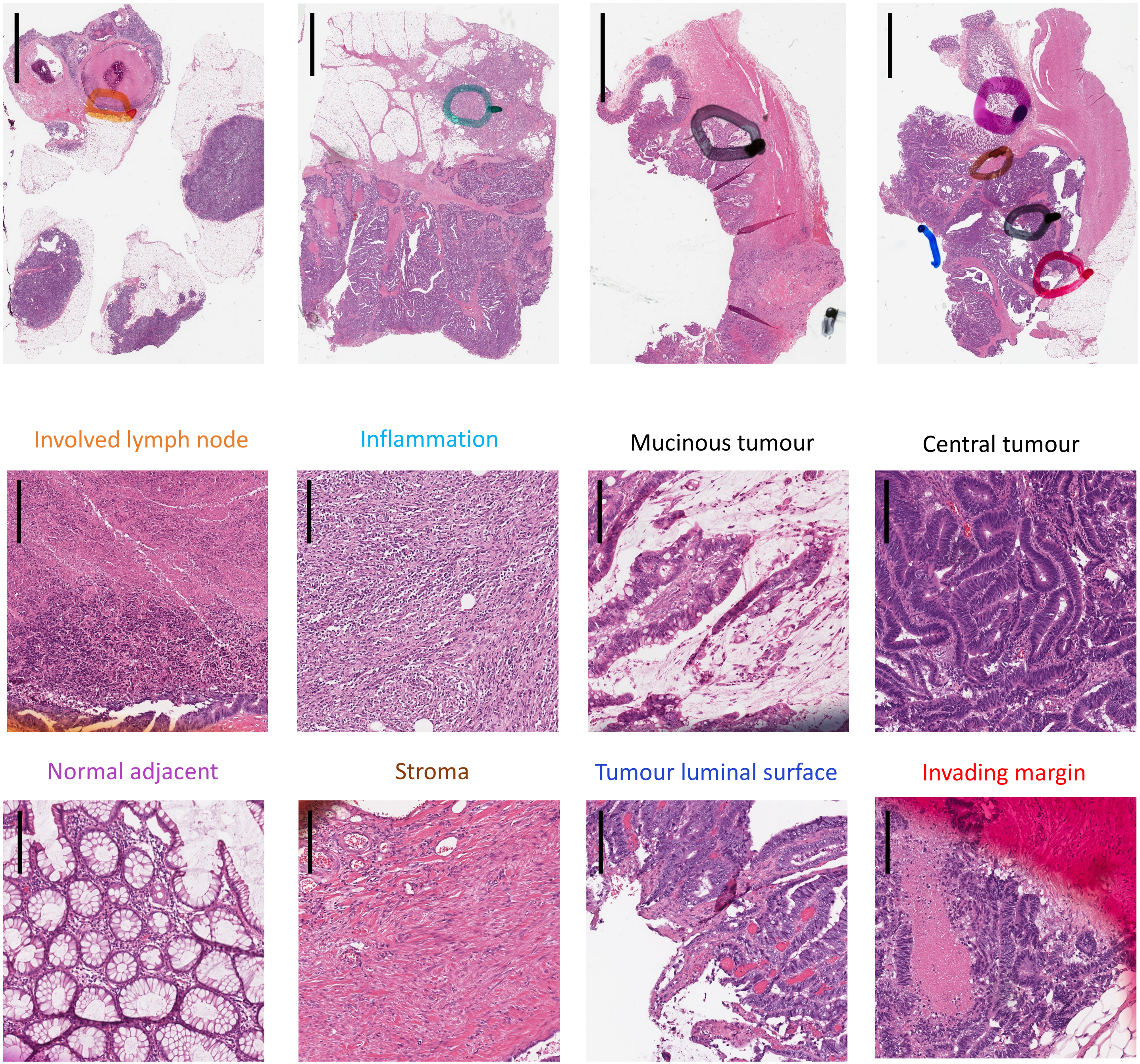
Involved lymph node
Inflammation
Mucinous tumour
Central tumour
Normal adjacent
Stroma
Tumour luminal surface
Invading margin
