# Supplementary figures and images for: Fusobacterium nucleatum and Bacteroides fragilis detection in colorectal tumours: Optimal target site and correlation with total bacterial load
Source: PLoS One. 2022 Jan 7;17(1):e0262416. doi: 10.1371/journal.pone.0262416 (PMC8740967; doi:10.1371/journal.pone.0262416)

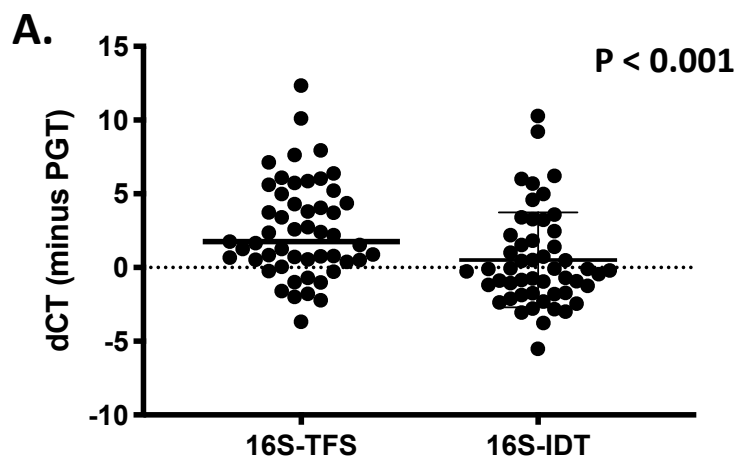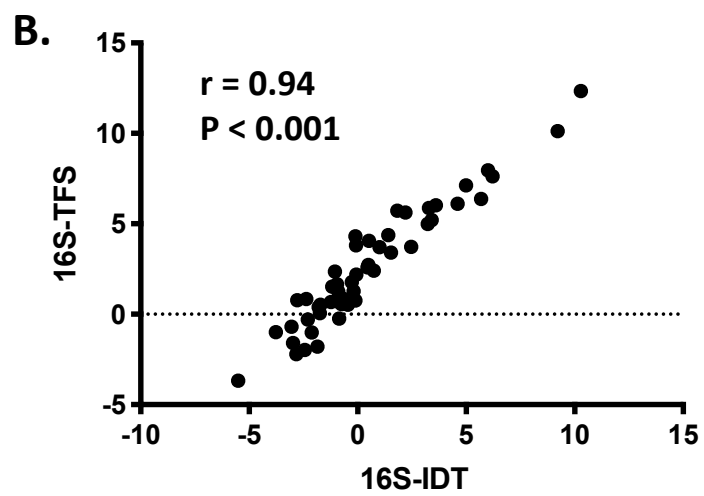

Supplement: S2 Fig — Relative amplification of 16S rRNA sequences using 16S-TFS primers (Thermo Fisher Scientific) and 16S-IDT primers (Integrated DNA Technologies, as published by Nadkarni et al 2002). (A) Groups compared using the Wilcoxon matched pairs test. Line at median. (B) Spearman’s Rho correlation analysis. (PDF) [file pone.0262416.s002.pdf]

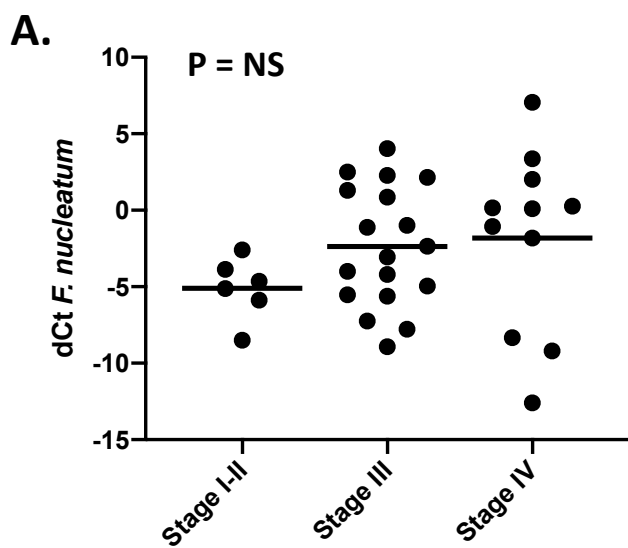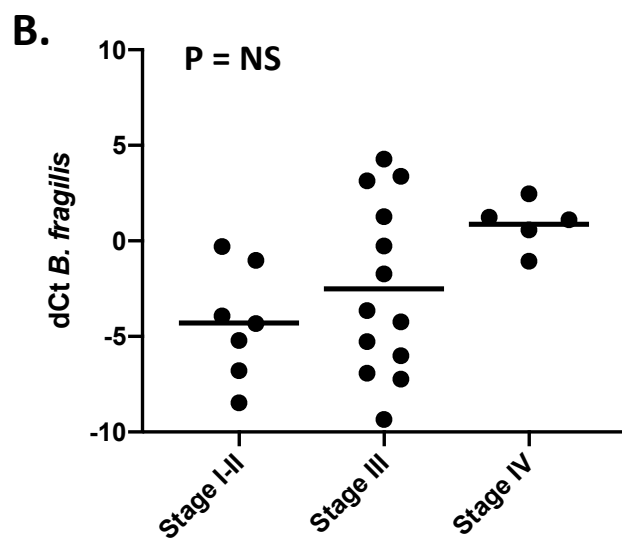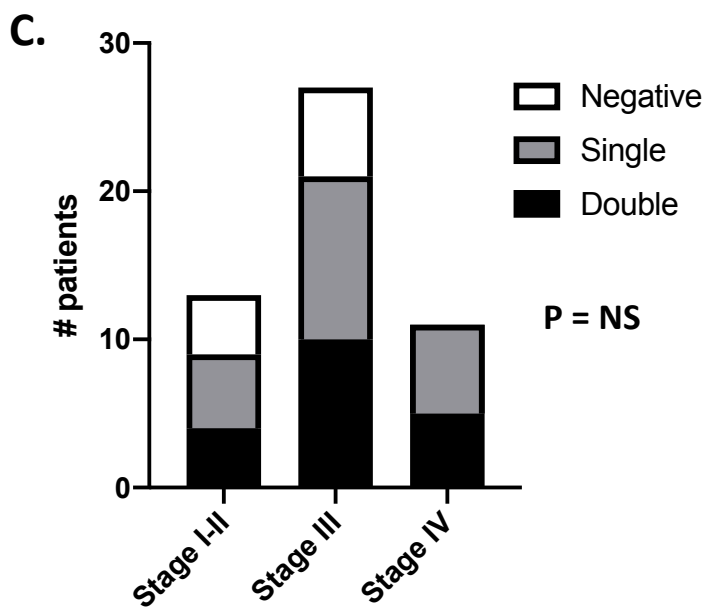

Supplement: S3 Fig — Relative expression (PGT—target) for F. nucleatum (A) and B. fragilis (B) at the TLS by disease stage, line at mean (ANOVA). C. Species positivity status at the TLS by disease stage (negative for F. nucleatum and B. fragilis vs single positive for F. nucleatum or B. fragilis vs double positive for F. nucleatum and B. fragilis), Fisher’s exact test. TLS, tumour luminal surface. (PDF) [file pone.0262416.s003.pdf]
