# Supplementary material for: Fusobacterium nucleatum and Bacteroides fragilis detection in colorectal tumours: Optimal target site and correlation with total bacterial load
Source: PLoS One. 2022 Jan 7;17(1):e0262416. doi: 10.1371/journal.pone.0262416 (PMC8740967; doi:10.1371/journal.pone.0262416)
Supplement: S1 Table — (PDF) [file pone.0262416.s004.pdf]

S1 Table Primer details

Target	Reference / Accession number	Primers/ Probe	Sequence (5'-3')
Fusobacterium nucleatum	Castellarin 2012	F<br>R<br>P	CAACCATTACTTTAACTCTACCATGTTCA<br>GTTGACTTTACAGAAGGAGATTATGTAAAAATC<br>TCAGCAACTTGTCCTTCTTGATCTTTAAATGAACC
Bacteroides fragilis (gyrase)	AB017713.1	F<br>R<br>P	ACGGCAGTTTCAAACACGAAA<br>TCGATGAAGCGTACAAATTCTCTT<br>TTTCTATTCGGAAGAGGGTT
Bacteroides fragilis toxin	U67735.1	F<br>R<br>P	TTAGTGCCCAGATGCAGGAT<br>CCGTCGTCATAACCTTCTGC<br>GCGGCGAACTCGGTTTATGCA
Bifidobacterium breve	AP012324.1	F<br>R<br>P	CCGGATTGATGTCTGCAGTGT<br>TCTTCACCGCCATGTATCCA<br>ACTTTCTGCCATACCGGATA
Campylobacter showae (chaperonin 60)	DQ059461.2	F<br>R<br>P	CTACGGTTTTGGCGCACTCT<br>GCGTTTTACTTCGACCGGATT<br>AAACATTACCGCAGGAGC
Leptotrichia buccalis	CP001685.1	F<br>R<br>P	GGTAAATCCGCGTGTCTAAATGT<br>TTTTCTTGGCAGTGTAGGATCTGTA<br>AGACCTGATGGGCAAGT
Prostaglandin transporter	Castellarin 2012	F<br>R<br>P	ATCCCCAAAGCACCTGGTTT<br>AGAGGCCAAGATAGTCCTGGTAA<br>CCATCCATGTCCTCATCTC

Castellarin et al. Fusobacterium nucleatum infection is prevalent in human colorectal carcinoma. Genome Research. 2012;22(2):299-306. doi: 10.1101/gr.126516.111. PubMed PMID: PMC3266037.
