# Supplementary material for: Fusobacterium nucleatum and Bacteroides fragilis detection in colorectal tumours: Optimal target site and correlation with total bacterial load
Source: PLoS One. 2022 Jan 7;17(1):e0262416. doi: 10.1371/journal.pone.0262416 (PMC8740967; doi:10.1371/journal.pone.0262416)
Supplement: S2 Table — (PDF) [file pone.0262416.s005.pdf]

S2 Table Species positivity status and total bacterial load according to clinicopathologic factors

		F. nucleatum			*B. fragilis*			16S		
		**Negative n (%)**	**Positive n (%)**	**P**	**Negative n (%)**	**Positive n (%)**	**P**	**Low n (%)**	**High n (%)**	**P**
Age	≤ 68	8 (33.3)	16 (66.7)	1.000	16 (66.7)	8 (33.3)	0.051	15 (62.5)	9 (37.5)	0.164
	> 68	8 (29.6)	19 (70.4)		10 (37.0)	17 (63.0)		11 (40.7)	16 (59.3)	
Gender	Male	10 (26.3)	28 (73.7)	0.299	20 (52.6)	18 (47.4)	0.755	20 (52.6)	18 (47.4)	0.755
	Female	6 (46.2)	7 (53.8)		6 (46.2)	7 (53.8)		6 (46.2)	7 (53.8)	
Tumour location	Right	6 (24.0)	19 (76.0)	0.394	11 (44.0)	14 (56.0)	0.578	11 (44.0)	14 (56.0)	0.578
	Left	7 (35.0)	13 (65.0)		11 (55.0)	9 (45.0)		11 (55.0)	9 (45.0)	
	Rectum	3 (50.0)	3 (50.0)		4 (66.7)	2 (33.3)		4 (66.7)	2 (33.3)	
Histological grade	Low	8 (25.8)	23 (74.2)	0.730	18 (58.1)	13 (41.9)	0.348	14 (45.2)	17 (54.8)	0.530
	High	5 (33.3)	10 (66.7)		6 (40.0)	9 (60.0)		9 (60.0)	6 (40.0)	
KRAS mutation	No	1 (6.7)	14 (93.3)	1.000	7 (46.7)	8 (53.3)	0.400	5 (33.3)	10 (66.7)	1.000
	Yes	1 (12.5)	7 (87.5)		2 (25.0)	6 (75.0)		2 (25.0)	6 (75.0)	
BRAF mutation	No	1 (5.9)	16 (94.1)	0.463	6 (35.3)	11 (64.7)	0.643	5 (29.4)	12 (70.6)	1.000
	Yes	1 (16.7)	5 (83.3)		3 (50.0)	3 (50.0)		2 (33.3)	4 (66.7)	

Groups compared using Fisher's Exact test
16S dCt split at the median value
23/51 patients underwent *KRAS*/*BRAF* mutation testing
