# Supplementary material for: Fusobacterium nucleatum and Bacteroides fragilis detection in colorectal tumours: Optimal target site and correlation with total bacterial load
Source: PLoS One. 2022 Jan 7;17(1):e0262416. doi: 10.1371/journal.pone.0262416 (PMC8740967; doi:10.1371/journal.pone.0262416)
Supplement: S3 Table — Percentage of total reads attributed to each taxon. (PDF) [file pone.0262416.s006.pdf]

S3 Table Diversity profiling results. Percentage of total reads attributed to each taxon

**Taxon**	**Patient 26**	**Patient 28**	**Patient 29**	**Patient 32**	**Patient 35**
Bacteroides fragilis	**42.0766%**	**36.8439%**	0.0000%	0.0000%	0.0000%
Lachnospiraceae family	0.2742%	**30.7839%**	2.2511%	**13.8034%**	**26.9970%**
Bacteroides genus	**10.0906%**	2.1301%	**17.0048%**	**29.4935%**	2.2245%
Fusobacterium genus	**10.5076%**	**12.1084%**	**25.4171%**	0.0993%	0.0000%
Parabacteroides genus	0.0000%	0.0000%	0.0445%	0.0000%	**24.6714%**
Ruminococcaceae family	1.4356%	0.9042%	3.2854%	**18.5204%**	0.0000%
Prevotella genus	0.0000%	0.0000%	**18.0367%**	0.0000%	**5.9656%**
Selenomonas genus	**13.7821%**	0.0000%	0.4446%	0.0000%	0.0000%
Sphingomonas genus	0.0000%	0.0000%	0.0000%	0.0000%	**11.7290%**
Campylobacter genus	1.2282%	0.0000%	**10.0761%**	0.0000%	0.0000%
Propionibacterium acnes	0.0000%	0.1188%	0.0000%	**5.4121%**	**5.5612%**
Parabacteroides gordonii	0.0000%	0.0000%	0.0000%	0.0000%	**10.2123%**
Rikenellaceae family	0.4839%	0.1710%	0.6997%	**7.7458%**	0.0000%
Lachnospiraceae other	0.0691%	0.8723%	1.2238%	0.3972%	**5.9656%**
Streptococcus genus	**7.8923%**	0.0000%	0.0351%	0.0000%	0.0000%
Veillonella dispar	0.0000%	**6.0861%**	0.0234%	0.0000%	0.0000%
Eikenella genus	4.7699%	0.9709%	0.0000%	0.0000%	0.1011%
Clostridiales order	0.4977%	0.0609%	1.7012%	0.6951%	2.4267%
Clostridium genus	0.0000%	**5.1036%**	0.0094%	0.0000%	0.0000%
Dorea genus	0.0253%	0.0000%	0.1123%	4.4191%	0.0000%
Unassigned	0.3249%	0.4753%	0.5476%	1.3903%	1.7189%
Porphyromonas endodontalis	0.0000%	0.0000%	4.1020%	0.0000%	0.0000%
Enterobacteriaceae family	0.6729%	0.0029%	3.3228%	0.0000%	0.0000%
Erysipelotrichaceae family	0.0807%	0.0174%	0.1287%	2.6316%	0.0000%
Parabacteroides distasonis	0.3111%	0.0000%	0.1872%	2.3337%	0.0000%
Bacteroides other	0.0000%	1.0057%	0.3346%	1.3903%	0.0000%
Lactobacillus genus	0.0000%	0.0000%	0.0000%	2.6812%	0.0000%
Megasphaera genus	0.0714%	0.0000%	0.0000%	2.5323%	0.0000%
Ralstonia genus	0.0000%	0.0000%	0.0000%	0.0000%	2.4267%
Lachnospira genus	0.0000%	0.0000%	0.0000%	2.1847%	0.0000%
Oxalobacteraceae family	0.0000%	0.0000%	0.0000%	1.8868%	0.0000%
Oscillospira genus	0.3526%	0.0493%	1.3970%	0.0000%	0.0000%
Clostridiales other	0.3894%	0.1536%	0.9220%	0.0497%	0.0000%
Dethiosulfovibrionaceae TG5 genus	0.0876%	0.0000%	1.2800%	0.0000%	0.0000%
Collinsinella stercoris	0.0000%	0.0000%	0.0000%	0.9930%	0.0000%
TM7-3 class	0.0000%	0.0000%	0.0000%	0.9930%	0.0000%
Barnesiellaceae family	0.0000%	0.8607%	0.1217%	0.0000%	0.0000%
Bacteriodes uniformis	0.1452%	0.0000%	0.3861%	0.3476%	0.0000%
Dialister genus	0.0000%	0.0000%	0.7348%	0.0000%	0.0000%
Phascolarctobacterium genus	0.6106%	0.0000%	0.0936%	0.0000%	0.0000%
Bilophila genus	0.5415%	0.1420%	0.0000%	0.0000%	0.0000%
Aeromonadaceae other	0.0000%	0.0000%	0.6716%	0.0000%	0.0000%
Mogibacterium genus	0.3986%	0.0000%	0.2223%	0.0000%	0.0000%
Blautia genus	0.3825%	0.0000%	0.2153%	0.0000%	0.0000%

S3 Table cont.

Taxon	Patient 26	Patient 28	Patient 29	Patient 32	Patient 35
Streptococcus anginosus	0.4954%	0.0000%	0.0679%	0.0000%	0.0000%
Sutterella genus	0.5415%	0.0000%	0.0000%	0.0000%	0.0000%
Dorea other	0.4447%	0.0000%	0.0749%	0.0000%	0.0000%
Ruminococcaceae other	0.0000%	0.0000%	0.4937%	0.0000%	0.0000%
Leptotrichia genus	0.0000%	0.0000%	0.4563%	0.0000%	0.0000%
Bacteroides caccae	0.3272%	0.0000%	0.1006%	0.0000%	0.0000%
Alphaproteobacteria RF32	0.0000%	0.0000%	0.3276%	0.0000%	0.0000%
Roseburia genus	0.0000%	0.3101%	0.0000%	0.0000%	0.0000%
Christensenellaceae family	0.0000%	0.0000%	0.2948%	0.0000%	0.0000%
Oribacterium genus	0.0000%	0.0000%	0.2691%	0.0000%	0.0000%
Treponema genus	0.0000%	0.0000%	0.2597%	0.0000%	0.0000%
Prevotella melaninogenica	0.0000%	0.0000%	0.2340%	0.0000%	0.0000%
Ruminococcus gnavus	0.0000%	0.1681%	0.0655%	0.0000%	0.0000%
Cyanobacteria 4C0d-2 YS2 order	0.0000%	0.2000%	0.0000%	0.0000%	0.0000%
Catonella genus	0.0000%	0.0000%	0.1942%	0.0000%	0.0000%
Akkermansia muciniphila	0.0000%	0.1942%	0.0000%	0.0000%	0.0000%
Ruminococcus genus	0.0000%	0.0000%	0.1895%	0.0000%	0.0000%
Bacteroidales order	0.0000%	0.0000%	0.1732%	0.0000%	0.0000%
Aeromonadaceae family	0.0000%	0.0000%	0.1708%	0.0000%	0.0000%
Desulfovibrio genus	0.0000%	0.0000%	0.1638%	0.0000%	0.0000%
Coprococcus genus	0.0668%	0.0000%	0.0913%	0.0000%	0.0000%
Prevotella other	0.1498%	0.0000%	0.0000%	0.0000%	0.0000%
Clostridiaceae family	0.0000%	0.0000%	0.1451%	0.0000%	0.0000%
Peptostreptococcaceae family	0.1267%	0.0000%	0.0000%	0.0000%	0.0000%
Schwartzia genus	0.0438%	0.0000%	0.0796%	0.0000%	0.0000%
Eubacterium biforme	0.0000%	0.0000%	0.1100%	0.0000%	0.0000%
Mogibacteriaceae family	0.0737%	0.0000%	0.0328%	0.0000%	0.0000%
Odoribacter genus	0.0000%	0.0840%	0.0164%	0.0000%	0.0000%
Desulfobulbus genus	0.0000%	0.0000%	0.0959%	0.0000%	0.0000%
Ruminococcus genus	0.0000%	0.0927%	0.0000%	0.0000%	0.0000%
Slackia genus	0.0899%	0.0000%	0.0000%	0.0000%	0.0000%
Treponema socranskii	0.0000%	0.0000%	0.0866%	0.0000%	0.0000%
Megamonas genus	0.0000%	0.0000%	0.0819%	0.0000%	0.0000%
Peptococcaceae rc4-4 genus	0.0000%	0.0000%	0.0796%	0.0000%	0.0000%
Butyricimonas genus	0.0000%	0.0000%	0.0702%	0.0000%	0.0000%
Haemophilus parainfluenzae	0.0000%	0.0000%	0.0702%	0.0000%	0.0000%
Clostridiaceae family	0.0000%	0.0000%	0.0679%	0.0000%	0.0000%
Peptostreptococcus genus	0.0000%	0.0000%	0.0655%	0.0000%	0.0000%
Porphyromonadaceae family	0.0000%	0.0638%	0.0000%	0.0000%	0.0000%
Collinsella aerofaciens	0.0253%	0.0000%	0.0257%	0.0000%	0.0000%
Blautia producta	0.0323%	0.0000%	0.0187%	0.0000%	0.0000%
Coriobacteriaceae family	0.0000%	0.0000%	0.0445%	0.0000%	0.0000%
Prevotella tannerae	0.0000%	0.0000%	0.0445%	0.0000%	0.0000%
Holdemania genus	0.0000%	0.0000%	0.0421%	0.0000%	0.0000%
Eggerthella lenta	0.0415%	0.0000%	0.0000%	0.0000%	0.0000%

S3 Table cont.

**Taxon**	**Patient 26**	**Patient 28**	**Patient 29**	**Patient 32**	**Patient 35**
Tenericutes RF3 ML615J-28 order	0.0000%	0.0000%	0.0398%	0.0000%	0.0000%
Prevotella copri	0.0369%	0.0000%	0.0000%	0.0000%	0.0000%
Bulleidia genus	0.0000%	0.0000%	0.0281%	0.0000%	0.0000%
Victivallaceae family	0.0000%	0.0261%	0.0000%	0.0000%	0.0000%
Mollicutes RF39 order	0.0000%	0.0000%	0.0257%	0.0000%	0.0000%
Synergistaceae other	0.0000%	0.0000%	0.0187%	0.0000%	0.0000%
Prevotella genus	0.0000%	0.0000%	0.0140%	0.0000%	0.0000%
Parvimonas genus	0.0000%	0.0000%	0.0140%	0.0000%	0.0000%
Gemella genus	0.0000%	0.0000%	0.0094%	0.0000%	0.0000%
Turicibacter genus	0.0023%	0.0000%	0.0047%	0.0000%	0.0000%
Adlercreutzia genus	0.0000%	0.0000%	0.0047%	0.0000%	0.0000%
Peptococcaceae family	0.0000%	0.0000%	0.0047%	0.0000%	0.0000%

Taxa representing ≥ 5% of total for each patient are highlighted bold
